# Effects of changing mosquito host searching behaviour on the cost effectiveness of a mass distribution of long-lasting, insecticidal nets: a modelling study

**DOI:** 10.1186/1475-2875-12-215

**Published:** 2013-06-26

**Authors:** Olivier JT Briët, Nakul Chitnis

**Affiliations:** 1 Department of Epidemiology and Public Health, Swiss Tropical and Public Health Institute, Basel, Switzerland; 2 University of Basel, Basel, Switzerland

**Keywords:** Behavioural resistance, Physiological resistance, LLINs, Open malaria, ITNs, Mosquito, Modelling, Insecticide

## Abstract

**Background:**

The effectiveness of long-lasting, insecticidal nets (LLINs) in preventing malaria is threatened by the changing biting behaviour of mosquitoes, from nocturnal and endophagic to crepuscular and exophagic, and by their increasing resistance to insecticides.

**Methods:**

Using epidemiological stochastic simulation models, we studied the impact of a mass LLIN distribution on *Plasmodium falciparum* malaria. Specifically, we looked at impact in terms of episodes prevented during the effective life of the batch and in terms of net health benefits (NHB) expressed in disability adjusted life years (DALYs) averted, depending on biting behaviour, resistance (as measured in experimental hut studies), and on pre-intervention transmission levels.

**Results:**

Results were very sensitive to assumptions about the probabilistic nature of host searching behaviour. With a shift towards crepuscular biting, under the assumption that individual mosquitoes repeat their behaviour each gonotrophic cycle, LLIN effectiveness was far less than when individual mosquitoes were assumed to vary their behaviour between gonotrophic cycles. LLIN effectiveness was equally sensitive to variations in host-searching behaviour (if repeated) and to variations in resistance. LLIN effectiveness was most sensitive to pre-intervention transmission level, with LLINs being least effective at both very low and very high transmission levels, and most effective at around four infectious bites per adult per year. A single LLIN distribution round remained cost effective, except in transmission settings with a pre-intervention inoculation rate of over 128 bites per year and with resistant mosquitoes that displayed a high proportion (over 40%) of determined crepuscular host searching, where some model variants showed negative NHB.

**Conclusions:**

Shifts towards crepuscular host searching behaviour can be as important in reducing LLIN effectiveness and cost effectiveness as resistance to pyrethroids. As resistance to insecticides is likely to slow down the development of behavioural resistance and *vice versa*, the two types of resistance are unlikely to occur within the same mosquito population. LLINs are likely cost effective interventions against malaria, even in areas with strong resistance to pyrethroids or where a large proportion of host-mosquito contact occurs during times when LLIN users are not under their nets.

## Background

Long-lasting insecticidal net (LLIN) distribution campaigns have played an important role in recent successes in malaria control [1]. LLINs protect against mosquito bites that mostly occur indoors during sleeping hours, coinciding with times when people are using LLINs. Furthermore, the nets have a protective ‘community effect’ by killing mosquitoes [2], thereby reducing the probability that the infected mosquito will survive the extrinsic incubation period and become infectious.

Resistance to pyrethroids in mosquito populations is increasing [3], threatening the effectiveness of pyrethroid-based interventions such as LLINs [4]. LLIN effectiveness is also threatened by shifts in biting behaviour, from nocturnal towards crepuscular, with mosquitoes actively host searching during the early morning and/or evening, when many LLIN users are not under their nets [5-7]. For example, Russell and colleagues [8] observed increased outdoor feeding after exposure to insecticide treated nets (ITNs) in Tanzania. Such a shift could occur simply ifmosquitoes are unsuccessful in finding a blood meal during their normal active host-searching period and if mosquitoes learn [9,10] and repeat behaviour that resulted in a blood meal The above causes of these shifts can be described as phenotypic plasticity [5]. It could occur if mosquitoes that search for hosts indoors during sleeping hours have a higher risk of being killed (by LLINs). If there is a genetic basis for the behaviour [11], pressure from LLINs could select for alleles that are associated with crepuscular biting behaviour, resulting in decreased exposure of mosquitoes to LLINs, and decreased effectiveness of LLINs over time. If these alleles are associated with certain (sub) species, this could lead to differential suppression of (sub) species [8,12].

In this paper, ‘behavioural resistance’ is defined as the behaviour-related ability to be unaffected by something. Such ability can, but does not need to be, acquired (or evolved) in response to pressure. Mosquitoes that tend to bite during times that LLIN users are not under their nets are less affected by LLINs than nocturnal host-searching mosquitoes, and thus have some ‘behavioural resistance’ against LLINs. However, mosquitoes that avoid contact with LLINs are not necessarily more behaviourally resistant than mosquitoes that do not if the contact-avoiding mosquitoes subsequently fail to find a blood meal or have a lower survival rate associated with crepuscular activity.

If host-searching behaviour varies in the vector population, this has implications for LLIN effectiveness. Molineaux and colleagues [13] show that with the assumption of non-uniform exposure to insecticides, vector control tools are much less effective than under the usual implicit assumption of uniform exposure. If all female mosquitoes in a population have the same stochastic probability of behaving a certain way each gonotrophic cycle, then mosquito potential exposure to LLINs is uniform. If, at the other extreme, each female always repeats her behaviour each gonotrophic cycle, her behaviour being determined before (genetically or phenotypically) or during the first gonotrophic cycle (through learning from experience), behaviour is fully determined and potential exposure is non-uniform. In the former fully ‘probabilistic’ case, each mosquito has *a priori* the same chance of surviving each gonotrophic cycle. In the latter, fully ‘determined’ case, those mosquitoes that never search for human hosts indoors during human sleeping hours are never exposed to LLINs, and thus their survival is not affected by LLINs. While behaviour is unlikely to be fully probabilistic, it is also unlikely to be fully determined. In reality, the degree of determinedness will lie somewhere in between these two extremes.

The proportion of mosquito-host encounters that occur indoors during sleeping hours in the absence of bed nets gives an indication of how strongly the mosquito population can be affected by LLINs. The more crepuscular and exophagic the vector population, the lower this proportion. This proportion, also called the ‘π_i_ value’ [14] or ‘π_s_ value’ [15], can also be defined as “…the proportion of normal exposure of unprotected humans lacking nets that occurs at times and places when net users would be protected by sleeping under them” [16]. If host-searching behaviour is fully determined, LLINs cannot reduce exposure to bites beyond the level that occurred during human sleeping hours prior to invention. In such a case, and if mosquitoes are killed by LLINs, the π_i_ value will be lower during intervention than prior to intervention. However, if there is some stochasticity in the behaviour, LLINs can potentially reduce exposure beyond the level occurring during human sleeping hours prior to intervention, because all mosquitoes are at risk of being killed by LLINs.

A previous modelling study [17] quantified the sensitivity of the effectiveness of a mass distributed LLIN batch to insecticide resistance, as measured in experimental hut studies^a^. In that study, the pre-intervention π_i_ was kept constant at a value of 0.75, and host-searching behaviour was assumed to be fully determined. This paper extends that previous study by incorporating sensitivity to assumptions about host-searching behaviour into the analysis.

## Methods

**Figure 1:**
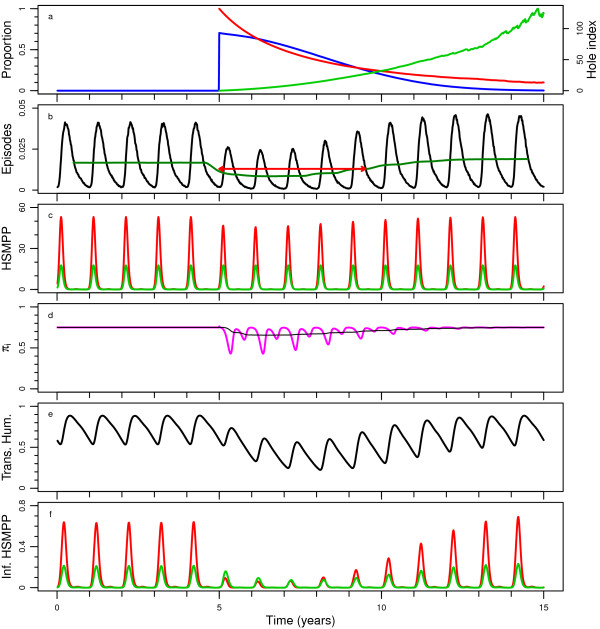
**Central scenario simulation with base model. a**) The blue line (on left vertical axis) represents the proportion of the population covered and the red line (on left vertical axis) represents the mean insecticide in the remaining LLINs as a proportion of its initial value. The light green line (on the right vertical axis) represents the mean hole index in the remaining LLINs [29]. **b**) The black line represents the number of episodes per person per five-day period, and the dark green line represents their one-year moving average. The red arrow indicates the approximate length of the effective lifetime of the LLIN distribution. **c**) Host-searching mosquitoes per person (HSMPP). The red line represents nocturnal mosquitoes and the lime green line represents crepuscular mosquitoes. **d**) The proportion of nocturnal mosquitoes out of the total host searching mosquitoes (π_i_) is represented by the magenta line. The thin black line represents the 365 day moving average. **e**) Proportion of humans transmitting malaria to mosquitoes. **f**) Infectious HSMPP. The red line represents nocturnal mosquitoes and the lime green line represents crepuscular mosquitoes.

The model used the same ‘central scenario’ as described earlier [17], with LLIN effect parameterization based on results from an experimental hut study with PermaNet 2.0 (P2) LLINs in Pitoa [18], where the *Anopheles gambiae* sensu lato population, consisting of 95% *Anopheles arabiensis* and 5% *Anopheles gambiae* sensu stricto, shows 70% mortality in 0.05% deltamethrin World Health Organization (WHO) insecticide susceptibility tests. Figure 1 illustrates the course of a central scenario simulation with a pre-intervention entomological inoculation rate (EIR) of 16 infectious bites per adult per annum (IBPAPA). In this scenario, LLINs are distributed to 70% of the simulated population in a single mass distribution in year five, after which they diminish in number following an attrition curve with a half-life of four years, and decay chemically (lose insecticide) and physically (form holes in their fabric), as shown in Figure 1a.

**Figure 2:**
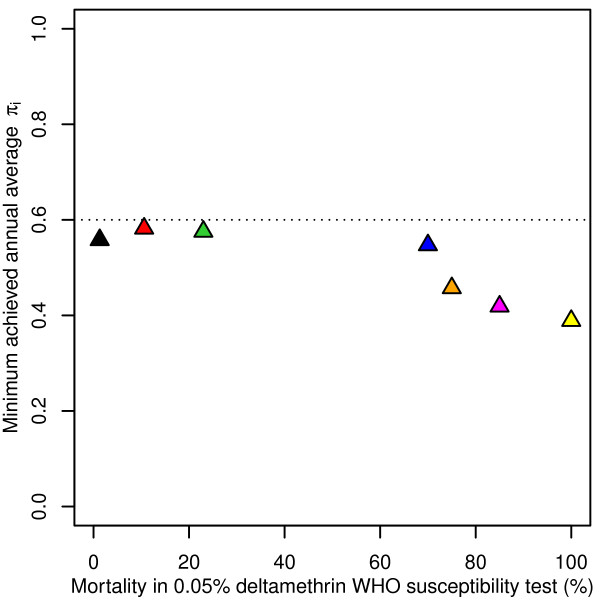
**Minimum achieved annual average π**_**i **_**during LLIN intervention.** Minimum achieved 365-day moving average π_i_ in simulations during intervention (cf. Figure 1d) with P2 mosquito nets assuming a pre-intervention π_i_ of 0.6 and fully determined host-searching behaviour, depending on susceptibility of the mosquito population, with black: ‘Akron’ [19], red: ‘Yaokoffikro’ [20], lime green: ‘Kou’ [18], dark blue: ‘Pitoa’ [18], orange: ‘Van Duc A’ [21], magenta: ‘Malanville’ [18], and yellow: ‘Zeneti’ [22]. The dotted horizontal line at π_i_ = 0.6 indicates the level of the pre-intervention indoor exposure when people would be protected by LLINs.

In this experiment, the pre- intervention π_i_ value varied between 0.6 (representing behaviourally-resistant mosquitoes) and 0.9 (representing behaviourally-susceptible mosquitoes), with 0.75 as the central value. The degree of determinedness in host-searching behaviour in the population was examined for the two extremes: fully probabilistic (each gonotrophic cycle, a mosquito may display a host-searching behaviour different from that of the previous gonotrophic cycle), and fully determined behaviour (a mosquito displays the same behaviour each gonotrophic cycle). The latter was modelled using two separate subpopulations, one of which was potentially affected by LLINs. The transmission intensity was varied over a wide range. Also, P2 LLIN effect parameterizations [17] were included in the experiment, for mosquito populations with a varying degree of physiological resistance to deltamethrin [18-22]. Under the assumption of fully determined host-searching behaviour, only the nocturnal adult host-searching population is directly affected by the LLINs (Figure 1c). In this scenario, host-searching behaviour is assumed to be non-genetically determined and mosquito emergence is assumed to be independent of the adult population size, thus the proportion of nocturnal mosquitoes (out of the total host-searching mosquitoes (π_i_)) is reduced temporarily and fluctuates strongly with the seasonal population dynamics driven mainly by seasonal fluctuations in breeding site capacity (Figure 1d). The amount of reduction in π_i_ depends not only on the coverage and quality of the LLINs, but also on the pre-intervention π_i_ itself and on the susceptibility of the mosquito population (Figure 2). Note that under an assumption of fully probabilistic behaviour, the π_i_ value during intervention remains equal to the value prior to intervention. The proportion of humans transmitting malaria to mosquitoes is also temporarily reduced (Figure 1e). The number of nocturnal infectious host-searching mosquitoes is strongly reduced by the LLINs, mostly as a result of the LLINs’ impact on their survival, reducing their longevity to less than the extrinsic incubation period (Figure 1f). The number of infectious crepuscular mosquitoes is also reduced, but to a lesser extent. This is exclusively due to the reduced transmission from humans to mosquitoes. During the first three years after LLIN distribution, transmission from crepuscular mosquitoes is more important than that from nocturnal mosquitoes.

For each scenario, ten simulations were run, each with a unique random seed, for each of 14 model variants [23] Simulations were run using the OpenMalaria modelling platform [23-25], which combines stochastic individual-based models for *Plasmodium falciparum* malaria in humans with a deterministic model for malaria in mosquitoes [25,26]. As was done previously [17], from each scenario output and based on the number of uncomplicated episodes, severe episodes, sequelae and deaths depending on age, disability adjusted life years (DALYs) and health system costs were calculated for each whole year during the simulation run. For intervention scenarios, the period of the epidemiological effect was determined. This was defined as the period from distribution of a batch of LLINs until the time that the effect of LLINs had waned to just over half of the maximum impact in terms of uncomplicated and severe episodes averted. This period, called the ‘effective lifetime’ of the batch of LLINs, is illustrated by the red arrow in Figure 1b. By comparing each scenario to its corresponding non-intervention scenario, the effectiveness of a mass distributed batch of LLINs was calculated in terms of episodes averted during the effective lifetime, and cost effectiveness in terms of net health benefits (NHB) [27], expressed in DALYs, depending on biting behaviour and pre-intervention EIR. The costs and cost savings to the health system are included in the NHB, using a ceiling ratio conversion factor of 235.28 United States $^2012^ per DALY. The method for calculating DALYs and NHB is described in detail by Briët and colleagues [17] in an additional file.

## Results

### LLIN effectiveness depending on host-searching behaviour and transmission level

**Figure 3:**
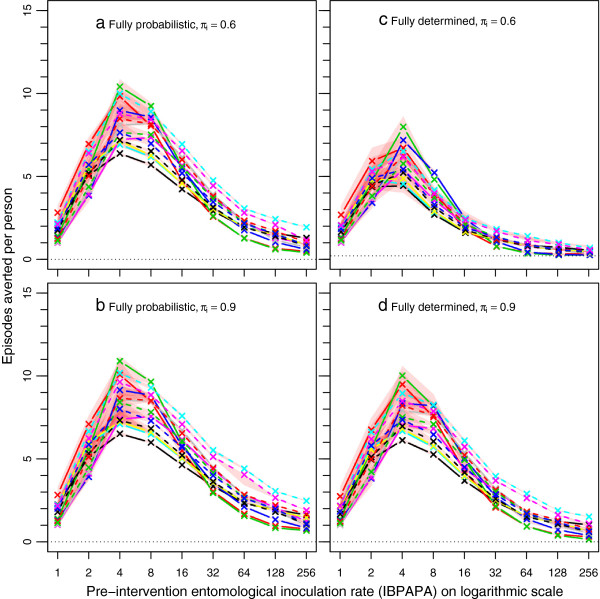
**Episodes averted by a mass LLIN distribution depending on host-searching behaviour.** Each line represents the median number of episodes averted per person of ten simulation runs (each with unique random seed) during the effective lifetime of a mass distribution, as compared to matching non-intervention scenarios, with population size = 100,000. The red semi-transparent polygons represent the range of the ten runs. Per panel, there are 14 lines (and 14 red polygons), each representing a malaria model variant. Model variants [28]: R0000 = solid black lines; R0063 = solid red lines; R0065 = solid green lines; R0068 = solid blue lines; R0111 = solid light blue lines; R0115 = solid magenta lines; R0121 = solid yellow lines; R0125 = solid grey lines; R0131 = dashed black lines; R0132 = dashed red lines; R0133 = dashed green lines; R0670 = dashed blue lines; R0674 = dashed light blue lines; R0678 = dashed magenta lines. **a**) Fully probabilistic host-searching behaviour, with a π_i_ value of 0.6. **b**) Fully probabilistic host-searching behaviour, with a π_i_ value of 0.9. **c**) Fully determined behaviour with a π_i_ value of 0.6. **d**) Fully determined behaviour with a π_i_ value of 0.9.

**Figure 4:**
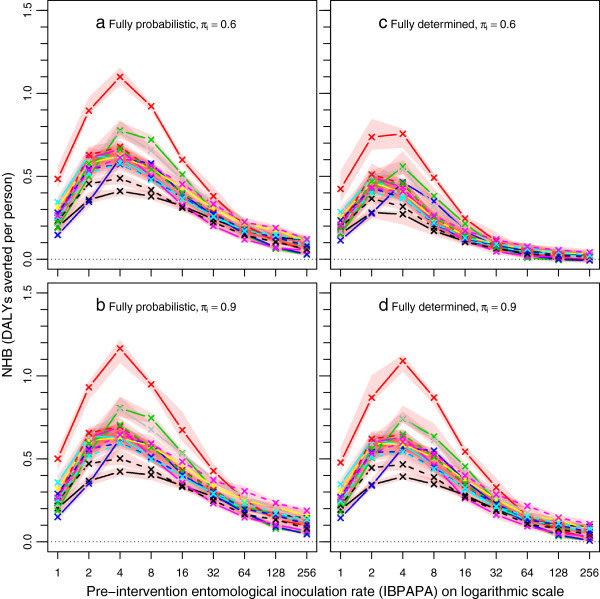
**Net health benefits of a mass LLIN distribution, depending on host searching behaviour.** Each line represents the median number of NHB, which are expressed in DALYs averted per person of ten simulation runs (each with unique random seed) during the effective lifetime of a mass distribution, as compared to matching non-intervention scenarios, with population size = 100,000. Legend further as in Figure 3.

The shape of the relationship between episodes averted (Figure 3) or NHB (Figure 4) and pre-intervention EIR appears to be similar for the four combinations of assumptions about host-searching behaviour, although with fully determined behaviour and a low π_i_ value of 0.6, LLINs are clearly less effective.

**Figure 5:**
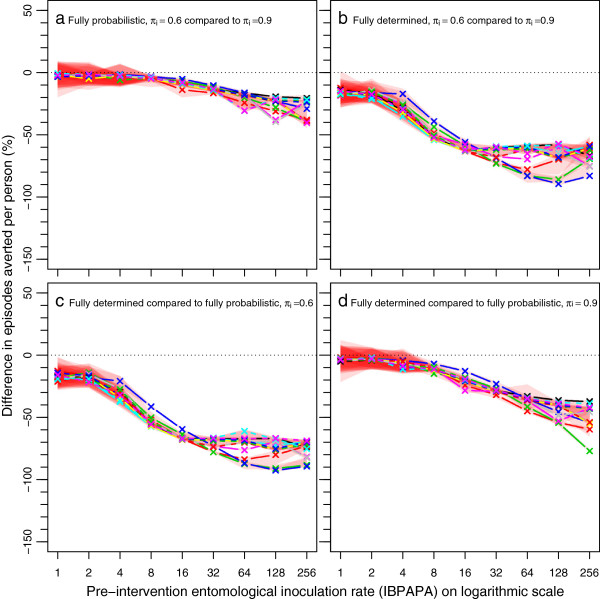
**Per cent differences in episodes averted by a mass LLIN distribution, depending on host-searching behaviour.** The per cent difference was calculated as 100×(A-B)/B, where A and B are host-searching behaviour assumptions. **a**) Fully probabilistic host-searching behaviour, with A is behaviour with a π_i_ value of 0.6 and B is behaviour with a π_i_ value of 0.9. **b**) Fully determined host-searching behaviour, with A is behaviour with a π_i_ value of 0.6 and B is behaviour with a π_i_ value of 0.9. **c**) Host-searching behaviour with a π_i_ value of 0.6, with A fully determined behaviour and B fully probabilistic behaviour. **d**) Host searching behaviour with a π_i_ value of 0.9, with A fully determined behaviour and B fully probabilistic behaviour. Horizontal dotted lines are at zero difference in episodes averted. Legend further as in Figure 3.

**Figure 6:**
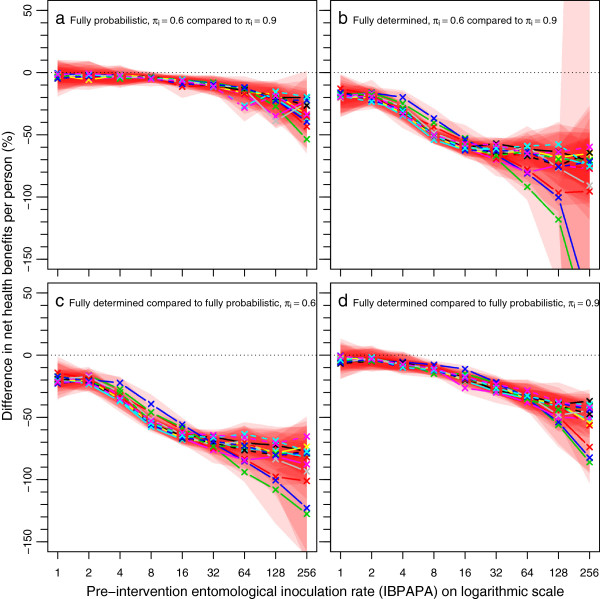
**Per cent differences in net health benefits averted by a mass LLIN distribution, depending on host-searching behaviour.** Legend as in Figure 5.

Figures 5 and 6 show the per cent differences of episodes averted and NHB, respectively, among the four combinations of assumptions about host-searching behaviour, depending on the pre-intervention EIR. The per cent difference increased non-linearly with increasing pre-intervention EIR. Figures S1 and S2 show absolute differences [see Additional file 1: Figures S1-S2], which are the proportional differences multiplied by the magnitude of the effect in the comparator.

When the behaviour was assumed to be fully probabilistic (Figures 5a and 6a), the difference between 90 or 60% of the mosquito-host contact occurring during times when LLIN users would not be under their nets (π_i_ values of 0.9 and 0.6, respectively) was much less pronounced (with a difference of up to about 30%) than when the behaviour was fully determined (with a difference up to about 70%) (Figures 5b and 6b). A comparison between the assumptions of fully probabilistic and fully determined behaviour at constant π_i_ value shows that at a π_i_ value of 0.6, the LLINs are much less effective with determined behaviour than with probabilistic behaviour (Figure 5c and Figure 6c). As expected, with a high π_i_ value of 0.9, this difference was much less pronounced (Figures 5d and 6d).

### Sensitivity analysis

**Figure 7:**
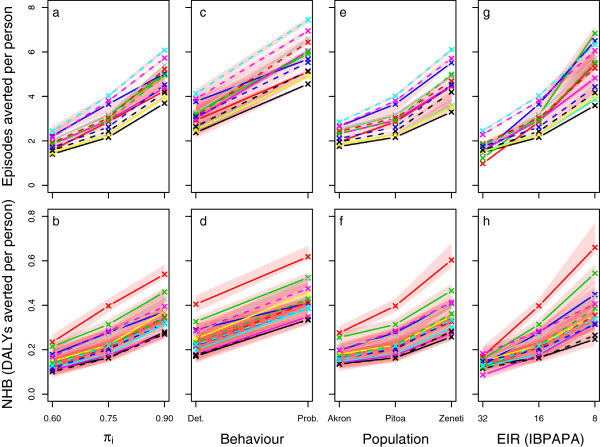
**Effectiveness of LLINs depending on host-searching behaviour, insecticide susceptibility, and transmission level.** Each line represents the median number of episodes averted per person (panels **a**, **c**, **e** &**g**) or NHB, which are expressed in DALYs averted per person (panels **b**, **d**, **f** &**h**) of ten simulation runs during the effective lifetime of a mass distribution, as compared to matching non-intervention scenarios. The red semi-transparent polygons represent the range of the ten runs. Per panel, 14 malaria model variant are shown with colour coding as indicated in the legend of Figure 3. In panels **a** &**b**, the π_i_ value was varied from 60 to 90 %, with a pre-intervention EIR of 16 IBPAPA, and susceptibility of mosquito population ‘Pitoa’. Host-searching behaviour was fully determined. In panels **c** &**d**, fully determined host-searching behaviour (Det.) is contrasted with fully probabilistic (Prob.) host-searching behaviour. The π_i_ value was 0.75, and other parameters were as in the first column of panels. In panels **e** &**f**, the susceptibility to pyrethroids is varied from the most resistant population, ‘Akron’, with less than 10.6 % mortality in 0.05% deltamethrin WHO susceptibility tests, to a medium susceptible population, ‘Pitoa’ (70% mortality), to a fully susceptible population, ‘Zeneti’ (100% mortality). The π_i_ value was 0.75, and other parameters were as in the first column of panels. In panels **g** &**h**, the pre-intervention EIR was varied between 32 and 8 IBPAPA for population ‘Pitoa’. The π_i_ value was 0.75, and other parameters were as in the first column of panels. Note that for each model variant, the middle value displayed is the same for all panels in a row of panels (except for panels **c** &**d**, where it corresponds to the left value).

Figure 7 illustrates the sensitivity of the effectiveness of a mass LLIN distribution to assumptions about host-searching behaviour around a central scenario and allows comparison with results from a previous analysis on sensitivity to insecticide susceptibility and pre-intervention EIR [17]. At a pre-intervention EIR of 16 IBPAPA, the proportion of mosquito-host interaction that, prior to intervention, occurred during times when people were indoors and asleep (Panels a & b) appears to be as important as insecticide resistance status (Panels e & f) if host-searching behaviour was assumed to be fully determined. As noted before [29], the pre-intervention EIR was extremely important. Even if varied over a small part of its potential range, the pre-intervention EIR had more impact on the effectiveness of an LLIN distribution than the π_i_ value and insecticide resistance of mosquito populations. Panels c & d show that (at a pre-intervention π_i_ value of 0.75) the effectiveness of a mass LLIN distribution is very sensitive to the assumptions about the degree of determinedness of the host-searching behaviour: If host-searching behaviour is fully probabilistic, the LLIN effectiveness is much larger. It should be noted that under the assumption of fully probabilistic behaviour, the effectiveness is much less sensitive to variations in the π_i_ value (see Figures 5a and 6a).

## Discussion

In this sensitivity analysis, a minimum pre-intervention π_i_ value of 0.6 was used. In the literature, π_i_ values for malaria vectors as low as 0.45 have been observed [8,16,30], but such low values were in areas already having high coverage of bed nets [30,31], and were likely low as a result of LLIN use or other ITNs [8]. Under the assumption of fully probabilistic host-searching behaviour, all mosquitoes have the same risk of being affected by LLINs and the π_i_ value does not change with LLIN deployment. However, under the assumption of fully determined behaviour, the LLINs killed only (a proportion of) those mosquitoes that searched during times when LLIN users were under their nets, thus the π_i_ value is brought down merely by selective killing of indoor nocturnal host-searching mosquitoes. With a pre-intervention π_i_ value of 0.6, the minimum annually smoothed π_i_ value varied between 0.39 (for the susceptible ‘Zeneti’ population) and 0.58 (for the resistant ‘Yaokoffikro’ population), depending on the mosquito population (Figure 2). This range includes those values observed in areas having high coverage of bed nets [8,16,30,31]. Assuming some degree of determinedness in host-searching behaviour, the pre-intervention π_i_ value (in the absence of LLINs) in these areas was likely higher [32], and a pre-intervention π_i_ value of 0.6 seems a reasonable lower limit to work with for primary malaria vectors.

In these simulations, the periodic emergence rate of new adult mosquitoes was independent of the number of adult mosquitoes [26]. This is under the assumptions that mosquito emergence is strongly dependent on breeding site capacity and that survival from egg to emerging adult is strongly regulated by density dependent processes. White and colleagues [33] show that at 50 to 80% LLIN coverage, these assumptions lead to only small underestimates in the effect of LLINs on the adult mosquito population, compared to models where the emergence rate is dependent on the number of adult mosquitoes (but where density dependent mechanisms still play an important role). Under the assumption of fully probabilistic or not genetically but otherwise determined mosquito host-searching behaviour, the assumption that the emergence rate of new adult mosquitoes is independent of the number of adult mosquitoes has no further consequences. However, if this behaviour is genetically determined (hereditary), higher survival of ‘behaviourally resistant’ mosquitoes (that bite during times that LLIN users are not under their nets) would lead to relatively more eggs being deposited with alleles associated with this trait (even under the assumption of random mating). Assuming no genetic disadvantage in the larval stage, this would lead to a larger proportion of ‘behaviourally resistant’ mosquitoes emerging and probably much lower π_i_ values than those observed in areas having high coverage of bed nets [8,16,30,31]. With continued selection pressure, alleles for crepuscular host-searching behaviour could rapidly spread through the mosquito population and eliminate the nocturnal mosquitoes unless the competition between them is weak [34], and the loss in LLIN cost effectiveness could be much higher than estimated by these models. However, such low π_i_ values have not (yet) been reported.

## Conclusions

The models were very sensitive to assumptions about the degree of the determinedness of mosquito host-searching behaviour when there was heterogeneity in this behaviour. This is in line with results from Molineaux and colleagues [13]. Under the assumption of fully probabilistic mosquito host-searching behaviour, the effectiveness of the LLINs appears relatively insensitive to the pre-intervention π_i_ values considered here^b^. Govella and colleagues [16] also use this assumption in a modelling study on the effect of the π_i_ value on the efficacy of ITNs. They, too, predict only limited effects, even with pre-intervention π_i_ values as low as 0.46. However, under the assumption of fully determined mosquito host-searching behaviour, LLIN effectiveness is much more sensitive to the pre-intervention π_i_ value. Although little is known about the degree of determinedness in mosquito host-searching behaviour, evidence that allele frequencies can be associated with outdoor-biting behaviour [11], and lower π_i_ values after exposure to ITNs or LLINs [8] suggests that it is not fully probabilistic. Estimation of LLIN effectiveness should therefore account for the likely non-uniform exposure of vectors [13] and more research on the degree of determinedness of host-searching behaviour in vector populations is warranted.

The sensitivity analysis (Figure 7) showed that shifts towards crepuscular host-searching behaviour, if this behaviour is determined, can reduce LLIN effectiveness as much as insecticide resistance in nocturnally biting mosquitoes can. However, the effect of the pre-intervention transmission level appears still more important than the effects of physiological and behavioural resistance on the effectiveness of LLINs. The models predicted that a single distribution of LLINs remains cost effective in the simulated scenarios, except for in transmission settings with a pre-intervention inoculation rate of over 64 annual infective bites and physiologically resistant mosquitoes (70% mortality in 0.05% deltamethrin WHO susceptibility tests) that displayed a high proportion (over 40%) of determined crepuscular host-searching behaviour, where some model variants showed negative NHB (Figure 4c). This is in line with results from a study during a period of increasing bed net use in São Tomé that suggests that bed nets reduced malaria prevalence [35], despite the vector being predominantly early and outdoor biting [36]. However, it should be noted that in most malaria-endemic countries, malaria control will not be limited to a single mass distribution of LLINs and cost-effectiveness may decrease with prolonged LLIN use.

Strong insecticide resistance and strong behavioural resistance against the same vector control intervention are unlikely to occur within the same population. Simulation results in Figure 2 illustrate that in the more insecticide-resistant populations (e.g., population ‘Akron’), nocturnally host-searching mosquitoes are less suppressed than in insecticide-susceptible populations (e.g., populations ‘Zeneti’ and ‘Malanville’). In this context, it is interesting that the sensitivity of LLIN effectiveness to host-searching behaviour (the π_i_ value) is weaker in populations that show insecticide resistance in WHO susceptibility tests as compared to fully susceptible populations [Additional file 2: Figure S3]. The two resistance types likely interfere with each other’s development: mosquitoes that are good at avoiding contact with insecticide do not need to develop physiological resistance and mosquitoes that are physiologically resistant do not need to avoid contact with the insecticide. Thus, on the one hand, survival of physiologically susceptible mosquitoes under insecticide pressure has been attributed to avoidance [37-39]. On the other hand, recently documented shifts^c^ in biting rhythm occurred in susceptible populations [8,40,41]. Even though the modelling results suggest that, for physiologically resistant mosquitoes with 30% survival in WHO susceptibility tests (population ‘Pitoa’) that also display determined crepuscular host-searching behaviour, LLINs might not be cost effective in very high transmission settings, evidence for the existence of such ‘double resistant’ malaria vector populations has yet to be found.

Although models predicted that the LLIN mass distribution would (still) be cost effective against malaria in areas with strong physiological resistance against pyrethroids or where a large proportion of host-mosquito contact occurs during times when LLIN users are not under their nets, in such situations, national malaria control programmes may consider complementing LLINs with other vector control interventions, such as those that are effective against crepuscular and exophagic mosquitoes in order to address the ‘residual’ transmission [6]. At higher transmission reduction targets than those obtainable with LLINs and case management, such combinations may be more cost effective.

### Endnotes

^a^These experimental huts studies measure effects of LLINs against indoor and nocturnally biting mosquitoes in terms of insecticidal effect and protection against feeding. These effects vary with mosquito population depending on physiological resistance in the population and the degree that mosquitoes from the population are deterred from entering the hut or dissuaded (repelled) from attacking once inside a hut with an LLIN, compared to a control hut. These last two types of mosquito behaviour, resulting in avoidance of contact with the insecticide, are also a form of behavioural resistance against being killed while host searching, just like shifts towards crepuscular host searching behaviour. Nocturnal avoidance of contact with insecticide treated material in otherwise physiologically susceptible mosquitoes [37] could be an important threat to the cost effectiveness of LLINs. Nevertheless, the population level protection against feeding and the population level insecticidal effect in the populations used for parameterization is highly correlated with mortality in WHO susceptibility assays [17], which only measure physiological or biochemical resistance.

^b^With π_i_ values closer to zero, not considered here, sensitivity would increase.

^c^Although lacking sufficient baseline data to document a shift, a study [42] in Punta Europa on Bioko Island, Equatorial Guinea found unusual high crepuscular and outdoor biting of *An. gambiae* sensu stricto after several years of indoor residual spraying with pyrethroids followed by several years of spraying with a carbamate insecticide. Although a high frequency of resistance against pyrethroids was found [43], this was not true for resistance against carbamates.

## Competing interests

The authors declare that they have no competing interests.

## Authors’ contributions

OJTB designed the experiments, analysed results and drafted the manuscript. NC helped conceive of the study and participated in the design. Both authors read and approved the final manuscript.

## Supplementary Material

Additional file 1: Figures S1-S2 Differences in episodes and net health benefits averted by a mass LLIN distribution, depending on host-searching behaviour. Click here for file

Additional file 2: Figure S3 Effectiveness of LLINs depending on host searching behaviour and insecticide susceptibility. Click here for file
