# Supplementary material for: Effects of changing mosquito host searching behaviour on the cost effectiveness of a mass distribution of long-lasting, insecticidal nets: a modelling study
Source: Malar J. 2013 Jun 26;12:215. doi: 10.1186/1475-2875-12-215 (PMC3706220; doi:10.1186/1475-2875-12-215)
Supplement: Additional file 1: Figures S1-S2 — Differences in episodes and net health benefits averted by a mass LLIN distribution, depending on host-searching behaviour. [file 1475-2875-12-215-S1.pdf]

Additional file 1

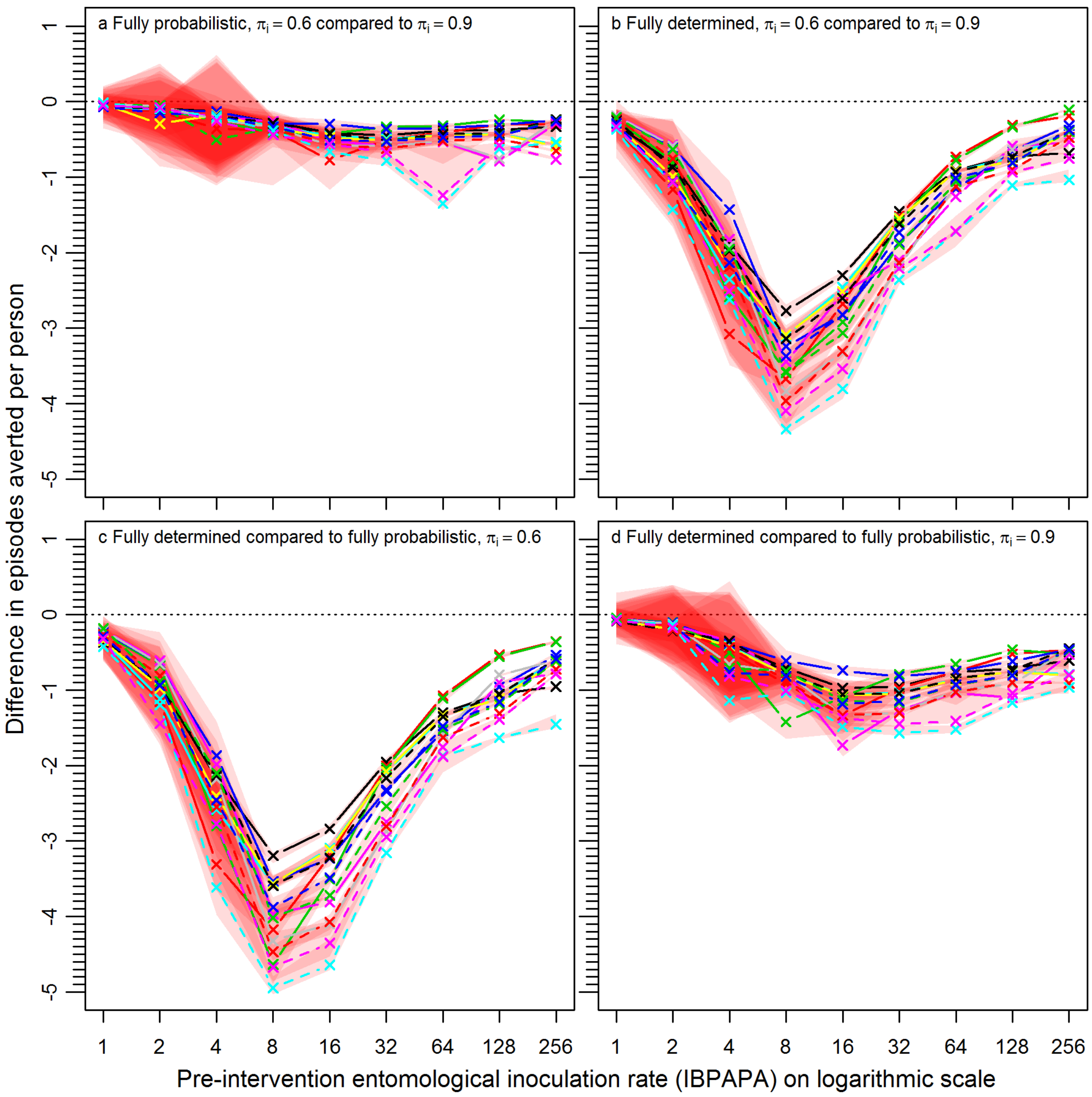

Figure S1 Differences in episodes averted by a mass LLIN distribution, depending on host-searching behaviour

The absolute difference was calculated as A-B, where A and B are host-searching behaviour assumptions. a) Fully probabilistic host-searching behaviour, with A is behaviour with a πi value of 0.6 and B is behaviour with a πi value of 0.9. b) Fully determined host-searching behaviour, with A is behaviour with a πi value of 0.6 and B is behaviour with a πi value of 0.9. c) Host-searching behaviour with a πi value of 0.6, with A fully determined behaviour and B fully probabilistic behaviour. d) Host searching behaviour with a πi value of 0.9, with A fully determined behaviour and B fully probabilistic behaviour. Horizontal dotted lines are at zero difference in episodes averted. Legend further as in Figure 3.

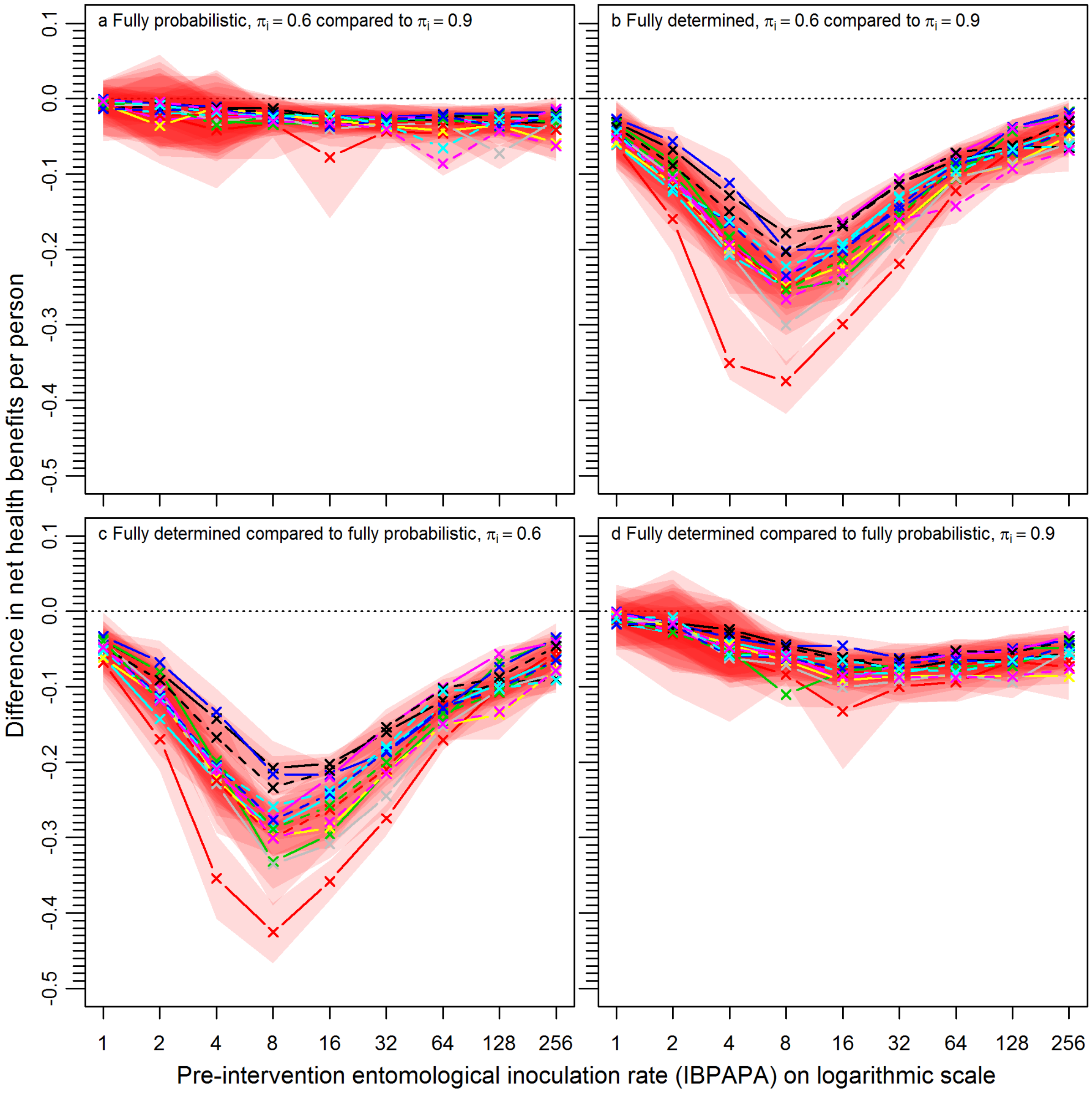

Figure S2 Differences in net health benefits averted by a mass LLIN distribution, depending on host-searching behaviour.

Legend as in Figure S1.
