# Supplementary material for: Effects of changing mosquito host searching behaviour on the cost effectiveness of a mass distribution of long-lasting, insecticidal nets: a modelling study
Source: Malar J. 2013 Jun 26;12:215. doi: 10.1186/1475-2875-12-215 (PMC3706220; doi:10.1186/1475-2875-12-215)
Supplement: Additional file 2: Figure S3 — Effectiveness of LLINs depending on host searching behaviour and insecticide susceptibility. [file 1475-2875-12-215-S2.pdf]

Additional file 2

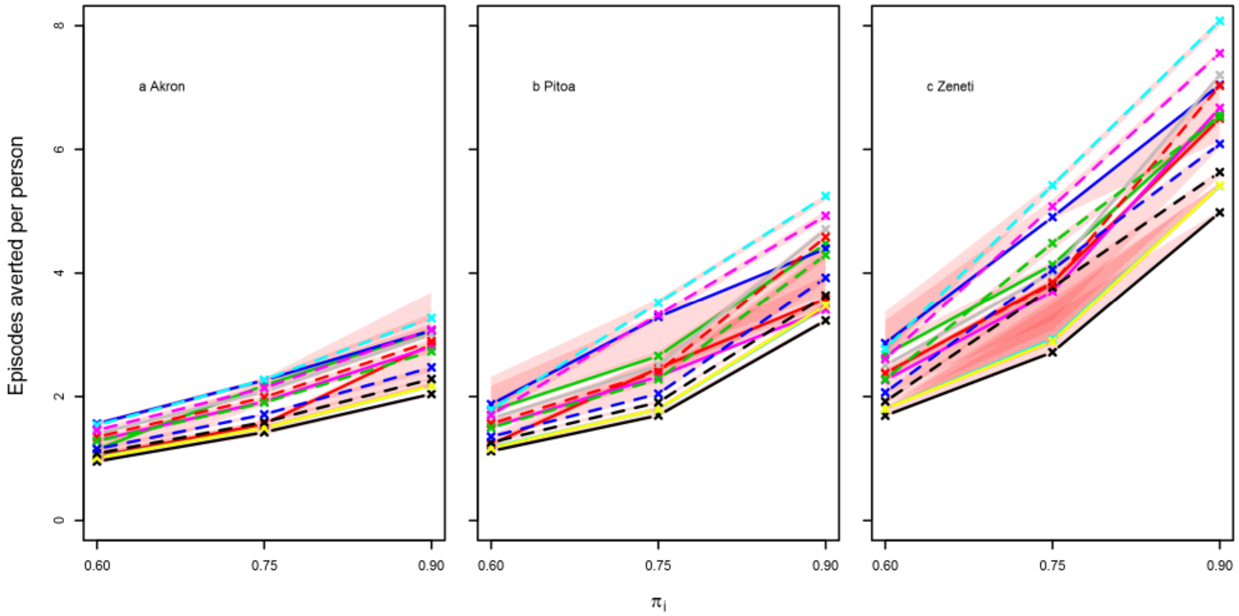

Figure S3 Effectiveness of LLINs depending on host searching behaviour and insecticide susceptibility. Each line represents the median number of episodes averted per person of 10 simulation runs (each with unique random seed) during the effective lifetime of a mass distribution, as compared to matching non-intervention scenarios. The red semi-transparent polygons represent the range of the 10 runs. Per panel, there are 14 lines (and 14 red polygons), each representing a malaria model variant, with colour coding as indicated in the legend of Figure 3. The proportion of the mosquito-host interaction that occurred during times when people were indoors and asleep (the π_i value) was varied from 60 to 90 %, with a pre-intervention entomological inoculation rate of 16 infectious bites per adult per annum. Host searching behaviour was fully determined (a mosquito displays the same behaviour each gonotrophic cycle). The susceptibility of mosquito population was that of 'Akron' (Panel **a**), with less than 10.6 % mortality in 0.05% deltamethrin WHO susceptibility tests, to a medium susceptible population, population 'Pitoa' (Panel **b**) (70% mortality), to a fully susceptible population, population 'Zeneti' (Panel **c**) (100% mortality).
